# DECADES OF CONNECTION: THE IMPACT OF SOCIAL NETWORK TRAJECTORIES ON COGNITIVE HEALTH ACROSS 29 YEARS

**DOI:** 10.1093/geroni/igae098.1163

**Published:** 2024-12-31

**Authors:** Rita Hu, Toni Antonucci, Kristine Ajrouch

**Affiliations:** University of Chicago, Chicago, Illinois, United States; University of Michigan, Ann Arbor, Michigan, United States; University of Michigan, Ann Arbor, Michigan, United States

## Abstract

The current study used Latent Growth Curve Modeling to examine the associations between overall network, closest, close and weak social tie trajectories across the lifespan and cognitive health later in life. Participants (N = 145) were from the longitudinal Social Relations study and the Detroit Area Wellness Network study, with four waves of data spanning from 1992 to 2021. Participants had a wide age range in Wave 1 (Mage (SD) = 46.9 (8.01) [37 - 71]) and were mostly women (69%) and White (82%). Social network structure was measured using hierarchical mapping with inner, middle, and outer circles symbolizing the emotional closeness of the relationships. Cognitive health was measured by a latent variable based on episodic memory, language, attention, and working memory. Results showed that increases in both overall network size and the size of closer social networks (middle circle) over time were significantly associated with better cognitive health (overall network size: β = 0.490, p <.064; middle circle size: β = 1.864, p =.005). Gender-specific analysis revealed no significant differences in model fit between constrained and unconstrained models, suggesting the absence of substantial gender differences. However, it was noted that for men, the increase in closer social networks over time positively impacts cognitive function. The study contributes to the understanding of the complex role social ties play in cognitive aging and underscores the necessity for targeted interventions to bolster social connections among older adults, with a nuanced approach considering gender-specific dynamics.

